# Molecular Scavengers, Oxidative Stress and Cardiovascular Disease

**DOI:** 10.3390/jcm8111895

**Published:** 2019-11-06

**Authors:** Alessandro Di Minno, Mariano Stornaiuolo, Ettore Novellino

**Affiliations:** Department of Pharmacy, University of Naples Federico II, 80131 Naples, Italy; mariano.stornaiuolo@unina.it (M.S.); ettore.novellino@unina.it (E.N.)

Cardiovascular disease (CVD) is the number one cause of deaths worldwide, with yearly deaths due to atherothrombosis—i.e., thrombosis complicating an atherosclerotic plaque—expected to increase from 17.3 to 23.6 million by 2030 [1]. Cardiovascular (CV) deaths account for approximately 1/3 of global deaths with medical expenses and medication costs associated with treatments totaling $126 billion in 2010 in the US [2]. Atherosclerosis—the underlying disease leading to atherosclerotic plaque formation and CV deaths—is multifactorial, major risk factors for it being type 2 diabetes mellitus, hypertension, smoking habit, overweight, and dyslipidemia. Early recognition and treatment of patients at high risk of atherosclerosis is a major goal to reduce the incidence of CV deaths. On the other hand, the increment (+12.5%) of CV deaths despite campaigns to combat tobacco use, high cholesterol and triglycerides, high blood pressure and high plasma glucose levels has fostered the search for additional mechanisms playing key roles in CV deaths [3]. 

A more in-depth analysis of the data reveals that in the years between 2005 and 2015, a 15.6% reduction of age-specific deaths had indeed occurred [4,5,6]. However, this was true in high-income countries (HICs)—where campaigns against risk factors for atherosclerosis had been implemented—but not in middle-income countries (MICs) and low-income countries (LICs) [5,7], such Countries accounting for the vast majority of CV deaths and, in turn, for the 12.5% increment. The latter information provides the background for the “25 × 25 Global Action Plan”, launched in 2013 by the World Health Organization and aimed at reducing mortality by 25% by 2025. This plan focuses on worldwide correction of tobacco use, diet, physical activity, and alcohol [8], the underlying philosophy being that early recognition and treatment of patients at high risk of atherosclerosis is a major goal to reduce the incidence of atherothrombotic events and CV disease. However, even taking this worldwide plan to combat established risk factors into account [9], compelling data argue for new directions (e.g., inflammation, gut microbiota, oxidative stress) to be pursued. Oxidative stress appears to play a key role in the onset and progression of the atherothrombotic process. Oxidative stress has been identified as a major regulatory mechanism for the endothelium, abnormally high levels of reactive oxygen species (ROS) overwhelming endogenous antioxidant systems, and leading to post-translational injury to proteins, lipids and DNA, i.e., to major cell functions [10]. 

The area of oxidative stress markers is increasing dramatically. In view of the accumulating evidence, and in order to identify newer directions to be pursued, a comprehensive state-of-the-art overview of the current knowledge in the area of oxidative stress is welcome. The comprehensive review [11] on the role of oxidative stress in CVD by Cervantes, Llanas-Cornejo and Husi from the Institute of Cardiovascular and Medical Sciences, University of Glasgow, published in the *Journal of Clinical Medicine* (https://www.mdpi.com/2077-0383/6/2/22) helps achieve this goal. Together with the wide spectrum of ROS involved in CVD, information is reported on an unfavorable glutathione (GSH, reduced form) and glutathione disulphide (GSSG, oxidized form) ratio (GSH/GSSG) in patients with acute myocardial infarction (AMI) [12] and on the relation between such unbalance and the progression of atherosclerotic lesions after percutaneous coronary interventions [13]. In the frame of the association of different oxidative stress markers with CVD [14,15], the ability of such markers to improve prediction of myocardial infarction, stroke and cardiovascular mortality by the European Society of Cardiology score is documented [16]. Drugs affecting major oxidative stress pathways and CVD treatment are also reported. In particular, the review describes molecules that play a role in the different stages of CV disease (e.g., sirtuin family activation) and proposes them as new potential drug approaches. 

It is also important to emphasize the role of this comprehensive review to identify directions to be explored for a better understanding of atherothrombosis and CVD. Together with the tight association between oxidative stress and CVD, in the last few years, the ability of exogenous antioxidant agents to prevent oxidative injury has been documented [17] (Figure 1). It was reported that oxidative injury by low-dose ionizing radiations in patients undergoing catheter ablation for cardiac arrhythmia is corrected by intravenous pretreatment with the potent antioxidant N-acetylcysteine, an acetylated cysteine residue able to maintain cysteine availability—the rate-limiting substrate for GSH re-synthesis—in the blood [18]. 

In concluding this editorial, we wish to call attention to the concept that, in a population at high risk of CVD (the PREDIMED study), a physiological antioxidant environment has been restored with food supplements [19]. Functional foods and nutraceuticals behave similarly, arguing for healthy balanced diets preventing oxidative stress and, conceivably, CVD in general. Oxidative stress may interfere with gut microbiota, a dynamic population of microorganisms (living in the human gastrointestinal tract) that produce a variety of hormone-like acting molecules, all able to strongly influence the physiology of the human host. Several investigators have focused their attention on trimethylamine (TMA), a metabolite originated from gut microbiota and further oxidized in the liver to trimethylamine N-oxide (TMAO), a nowadays accepted CVD risk factor [20]. Recent evidence indicates that ROS and oxidative stress are strictly related to the oxidation of TMA into TMAO [21]. Restoring a correct antioxidant endogenous environment not only may exert direct effects on endogenously produced antioxidants and increase their circulating levels, [22] but may also indirectly preserve the human-gut microbiota symbiosis, thus protecting the identity of gut derived metabolite and/or influencing their circulating levels. A reduction in blood levels of TMAO has been achieved in healthy and obese subjects by increasing circulating levels of resveratrol and polyphenols (both potent anti-oxidants) upon an 8-week treatment with a grape pomace extract [23].

All in all, continuous advances in science and new techniques are unraveling the complex etiology of CVD, allowing to identify the network of different factors interacting cumulatively to trigger the onset of the atherosclerotic phenomenon as well as its progression, and the need of innovative pharmacological approaches and new drug targets to prevent and treat it thoroughly. 

## Figures and Tables

**Figure 1:**
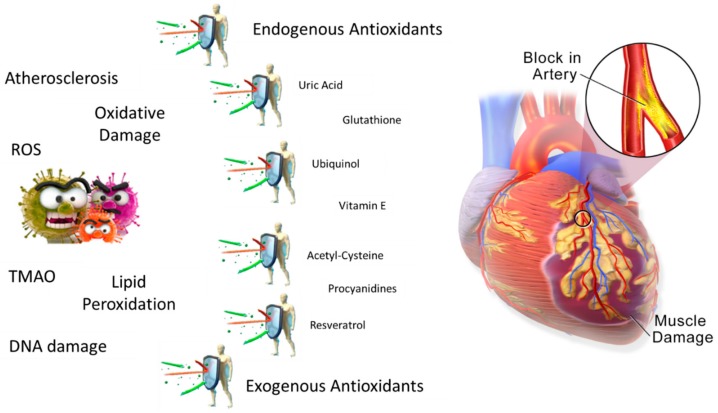
Schematic representation of the antioxidant mechanism of action pursued to avoid cardiovascular disease (CVD) onset. If the antioxidant system is unable to scavenge a high amount of reactive oxygen species (ROS), foods or nutraceuticals with proven antioxidant capacity should be provided.
